# Predictive value of thymidylate synthase for the prognosis and survival of lung adenocarcinoma patients

**DOI:** 10.3892/ol.2014.2658

**Published:** 2014-11-03

**Authors:** YAN HUANG, XIUHUA GUO, HONGYANG WANG, TIENIAN ZHU

**Affiliations:** 1 Department of Oncology, Hebei Medical University, Shijiazhuang, Hebei 050017, P.R. China; 2 Hebei United University Affiliated Hospital, Tangshan, Hebei 063000, P.R. China; 3 Department of Oncology, Bethune International Peace Hospital, Shijiazhuang, Hebei 050000, P.R. China

**Keywords:** lung adenocarcinoma, thymidylate synthase, prognosis, survival rate

## Abstract

Chemotherapy represents an important treatment modality for lung adenocarcinoma. Thymidylate synthase (TS) is an essential enzyme in DNA synthesis, and its overexpression has been associated with reduced sensitivity to antifolate agents. The aim of the current study was to investigate the expression of TS and the effect on prognosis in lung adenocarcinoma patients. Adenocarcinoma and adjacent carcinoma tissues were resected from 100 patients with lung adenocarcinoma and the TS levels were detected by immunohistochemical analysis. The values for overall survival (OS) and disease-free survival (DFS) were determined using the Kaplan-Meier analysis. The results indicated that the TS protein was expressed predominantly in adenocarcinoma tissues, which exhibited higher TS expression compared with the adjacent tissues (P<0.001). The statistical analysis indicated that TS expression was associated with the clinical stage and history of smoking (P<0.05). The Kaplan-Meier analysis results indicated that the DFS and OS in patients with high TS expression levels were significantly shorter compared with those with low expression levels (P<0.05). In conclusion, the results from this study suggested that TS may serve as an independent predictive factor for survival rate, which may indicate the prognosis of lung adenocarcinoma patients.

## Introduction

Adenocarcinoma is the most common type of lung cancer (1). Approximately 40% of lung cancers are adenocarcinomas, which usually originates from the peripheral lung tissue (2). Chemotherapy is an important therapeutic modality in the treatment of lung adenocarcinoma, however, the efficacy of the currently available therapy is limited (1). The enzyme, thymidylate synthase (TS), catalyzes the intracellular reaction that provides the sole de *novo* intracellular source of deoxythymidine monophosphate, or thymidylate, which is required for DNA replication and repair (4,5). The TS protein and messenger RNA levels are elevated in a number of human cancers (6) and have been found to correlate with poor prognosis in patients with colorectal (8), breast (9), head and neck (10) and pancreatic cancer. Thus, TS is considered as an oncogene and is the cellular target of chemotherapy drugs, 5-fluorouracil (12) and pemetrexed (13) The present study investigates the correlation between prognosis and TS expression in lung adenocarcinoma.

## Materials and methods

### Samples

Tumor and corresponding normal lung tissue was completely resected from 100 lung adenocarcinoma patients in 2009 at Hebei United University Affiliated Hospital (Tangshan, Hebei, China). The tissues were consecutively collected, and the immediately adjacent section was fixed in formalin, embedded in paraffin and subsequently used for hematoxylin and eosin staining and immunohistochemical analyses. None of the patients received pre- or post-operative treatment with chemotherapy or radiotherapy, according to an institutional treatment policy implemented at the time of specimen collection. Smokers were defined as current or former smokers. The median survival time was 40.18 months, with a minimum follow-up time of five years. The study was approved by the Institutional Review Board of Hebei United University. Written informed consent was obtained from all patients.

### Immunohistochemistry

Expression levels of the TS protein were detected by rabbit monoclonal anti-human thymidylate synthetase antibody (1/100 dilution; Boshide Biotechnology Co. Ltd., Wuhan, China) with overnight incubation at 4°C, followed by incubation with monoclonal goat anti-mouse horseradish peroxidase-conjugated secondary antibody at room temperature for 30 min (BA1051 against TS, 1:100 dilution, Boshide Biotechnology Co. Ltd.). Immunoreactions were revealed by a biotin-free, dextran chain-based detection system (Envision, DakoCytomation, Glostrup, Denmark) and developed using diaminobenzidine as the chromogen. Prior to the primary antibody incubation, the antigen retrieval was performed using the Pascal pressure chamber (Dako), heating in an ethylenediaminetetraacetic acid buffered solution (pH 8.0; Sigma-Aldrich, St. Louis, MO, USA) for 5 min at 125°C. The slides were counterstained with hematoxylin. For each tumor, the percentage expression of TS was evaluated.

### Quantitative polymerase chain reaction (qPCR)

Relative cDNA quantification for the TS gene, using the homo-GAPDH primer and probe (93bp; Invitrogen Life Technologies, Carlsbad, CA, USA) and the Homo-TS primer and probe (60bp; Invitrogen Life Techonologies) as the internal reference gene, was performed using a fluorescence-based quantitative detection method (ABI Prism 7700 Sequence Detection System; Applied Biosystems, Foster City, CA, USA). The sequences of the primers and probes used for TS and GAPDH have been published previously (14). All primers and probes (93bp; Invitrogen Life Technologies) were intron-spanning to avoid genomic DNA contamination (13). In each assay (96 wells), each sample was run in triplicate. The PCR mixture and cycling conditions were as previously described (15). Briefly, a PCR master mix, was aliquoted to each sample tube to a final volume of 45 μl. The PCR reaction mix consisted of: 2× TaqMan Universal PCR Master mix (25 μl), 260 nM (5 μl), 280 nM (5 μl). PCR analysis was performed using Taq polymerase (Invitrogen Life Technologies) and the reaction conditions were as follows: 95°C for 15 sec and 60°C for 1 min. A comparative Ct method, as previously described by Livak (16), detected relative gene expression. The ΔΔCt TS range of samples was determined by calculating the expression 2^−ΔΔCT^.

### Statistical analysis

Data were analyzed using SPSS 19.0 (IBM, Armonk, NY, USA) and are presented as the mean ± standard deviation. The values of overall survival (OS) and disease-free survival (DFS) were determined using the Kaplan-Meier analysis. Spearman’s rank analysis was also performed to determine the correlation. P<0.05 was considered to indicate a statistically significant difference.

## Results

### Clinical characteristics for 100 patients with lung adenocarcinoma

A total of 100 patients with lung adenocarcinoma were included in the present study. Overall, 33 cases were in clinical stage I, 42 cases were in stage II and 25 cases were in stage III. In total, 43 patients exhibited lymph node metastasis, while lymph node metastasis was not identified in the remaining 57 patients. The additional clinicopathological characteristics of the cases are also shown in Table I.

### TS expression in lung adenocarcinoma and adjacent carcinoma tissues

**Figure 1:**
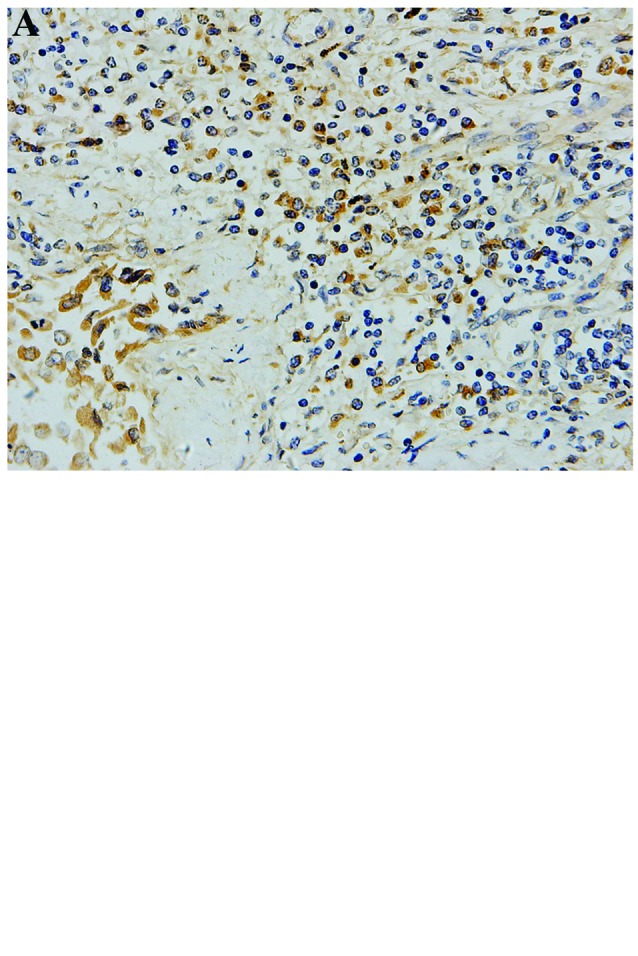
Expression of TS in adenocarcinoma tissues and adjacent carcinoma tissues, as observed by immunohistochemical evaluation (SP; ×400 magnification). (A) TS expression in adenocarcinoma tissues and (B) TS expression in adjacent carcinoma tissues. TS, thymidylate synthase; SP, streptavidin-peroxidase.

The changes in TS expression for each tissue type examined are shown in Table II. Immunohistochemical analysis indicated that the expression of TS was highly variable in the adenocarcinoma and adjacent carcinoma tissues (Fig. 1). The results presented in Table III confirmed that 43% of adenocarcinoma tissues exhibited a higher TS expression rate and 57% exhibited a lower expression rate. However, in the adjacent tissues, 12% exhibited a higher expression rate and 88% exhibited a lower expression rate. TS was expressed to a greater extent in the adenocarcinoma tissues, with significantly higher TS expression observed compared with the adjacent tissues (Fig. 1; Table II; P<0.001).

### Correlation between TS expression and the clinicopathological parameters

The statistical analysis indicated that TS expression correlated with the clinical stage and history of smoking (P<0.05), but was not associated with gender, age or the primary lesion in the lung adenocarcinoma tissues (P>0.05). The positive expression rate in stage III was significantly higher compared with that of stages I and II, and the level of expression in stage II was markedly higher compared with that of stage I in the lung adenocarcinoma tissues (Table III; P<0.05). The results also indicated that the TS protein expression level increased with increasing clinical stage, but not with age, gender, size of tumor and history of smoking (Table III; P>0.05).

### Effects of TS expression on the DFS and OS in lung adenocarcinoma patients

**Figure 2:**
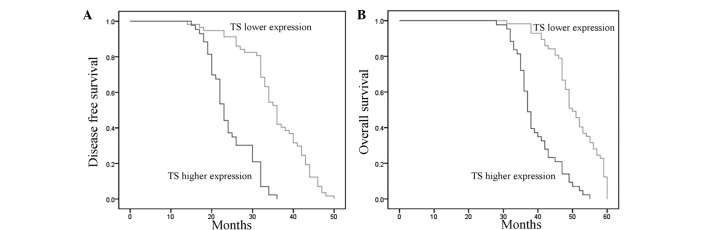
Overall and disease-free survival of lung adenocarcinoma patients were analyzed using the Kaplan-Meier method. (A) Disease-free and (B) overall survival of lung adenocarcinoma patients. TS, thymidylate synthase.

The Kaplan-Meier analysis results indicated that the mean DFS time was 23 months (95% CI, 21.72–24.27) in the tissues with high TS expression and 36 months (95% CI, 34.17–37.82) in the tissues with low TS expression, indicating a significant difference in the DFS between the tissues with high and low expression levels (Fig. 2A; P<0.05). The mean OS time was 37 months (95% CI, 35.57–38.43) in the tissues with high TS expression and 50 months (95% CI, 47.53–52.47) in the tissues with low TS expression, also demonstrating a significant difference in the OS between the tissues with high and low TS expression (Fig. 2B; P<0.001).

## Discussion

Lung cancer is a disease that is characterized by uncontrolled cell growth in the lung tissues. Lung cancer results in 1.38 million mortalities annually and is the most common cause of cancer-related mortalities in males and females globally (17). The most common types of lung cancer are small-cell lung carcinoma (SCLC) and non-SCLC (NSCLC; 80–85%). The most frequently observed symptoms are coughing (including hemoptysis), weight loss, shortness of breath and chest pain (18). Overall, 15% of patients diagnosed with lung cancer in the USA survive for five years after the diagnosis (19). Therefore, the study of prognostic biomarkers has attracted much attention with regard to lung cancer therapy.

Usually, the expression of TS and Src kinase is extremely low in normal tissues, however, the expression level is significantly increased in certain tumor tissues, including those from gastric (20), colorectal (21), urinary bladder (22) and mammary (23) cancer. It has been hypothesized that TS protein expression may be used as a prognostic biomarker for the tumors, however, the correlation between TS expression and lung cancer has been seldom studied (24). Therefore, the current study explored the significance of TS expression for lung adenocarcinoma patients.

Takezawa *et al* (25) reported that TS is overexpressed in certain tumors and that it may serve as a potential therapeutic target. Yang *et al* (26) found that TS gene expression was significantly increased in pemetrexed-resistant cells, and in a dose-dependent manner in pulmonary adenocarcinoma. The aforementioned conclusion is consistent with the results obtained in the present study. All the results indicated that increased expression and activity of TS in adenocarcinoma tissues may be associated with rapid tumor growth. The present study detected TS expression in adenocarcinoma tissue and adjacent carcinoma tissues by immunohistochemical assay, and analyzed the correlation between TS expression and clinical stage, gender, age, lymph node metastasis, history of smoking and primary lesion. The results indicated that TS expression correlated with clinical stage and history of smoking (P<0.05). Hashimoto *et al* (27) also examined TS gene expression using reverse transcription-qPCR, and the results indicated that TS expression correlated with the stage of disease, lymph node metastasis, tumor differentiation, prognosis and tumor cell proliferation. This result also agreed with the results observed in the present study, and demonstrated that TS protein expression may indicate the prognosis of lung adenocarcinoma. The present study also illustrated that the TS expression rate correlated with smoking, which may be due to the oxidative damage to the cells that is caused by smoking.

The results from the Kaplan-Meier analysis indicated that the DFS and OS times in the patients with high TS expression in the tissues were significantly shorter compared with those of with lower expression. Shimokawa *et al* (28) identified strong TS expression as an independent factor for tumor recurrence, and TS expression was associated with a poorer DFS, according to the survival analysis. Nakagawa *et al* (29) revealed that the five-year survival rates of a group with low expression levels of TS were significantly higher compared with the group with high expression levels, and concluded that the immunohistochemical evaluation of TS expression may be useful in predicting survival following the complete resection of lung adenocarcinomas. The results obtained in the current study also indicated the similar conclusion that TS expression in the adenocarcinoma tissues may serve as a prognostic marker for the survival rate.

In conclusion, the expression of TS was significantly increased in the adenocarcinoma tissues, and was markedly higher, compared with the adjacent carcinoma tissues. This indicated that TS may participate in the occurrence of lung adenocarcinoma. A correlation was identified between TS expression and differentiation, clinical stage and lymph node metastasis, which indicated that TS may participate in the progression of lung adenocarcinoma. TS expression was also observed to be an independent factor for survival rate, indicating that TS expression may be used to predict the prognosis of lung adenocarcinoma patients.

## Figures and Tables

**Table I:** Distribution of clinicopathological characteristics in 100 patients with lung adenocarcinoma.

Variables	n
Gender	
Male	55
Female	45
Age, years	
<60	61
≥60	39
Clinical stage	
I	33
II	42
III	25
Lymph node metastasis	
Yes	43
No	57

**Table II:** TS protein expression in lung adenocarcinoma and carcinoma adjacent tissues.

	TS expression, n				
				
Group	High expression	Low expression	Total, n	χ^2^	P-value
Adenocarcinoma tissues	43	57	100	24.1	<0.001
Adjacent tissues	12	88	100		

TS, thymidylate synthase.

**Table III:** Association between the expression of TS and the clinicopathological parameters in lung adenocarcinoma.

		TS				
				
Group	Cases, n	Positive, n	Negative, n	Positive rate, %	χ^2^	P-value
Gender					0.30	0.58
Male	55	25	30	45.5		
Female	45	18	27	40.0		
Age, years					1.30	0.25
<60	61	29	32	47.5		
≥60	25	35.9	39	14		
Clinical stage					23.50	0.00**
I	33	8	25	24.2		
II	42	14	28	33.3		
III	25	21	4	84.0		
Smoking					5.80	0.02*
≤400	56	30	26	53.6		
>400	44	13	31	29.5		
Primary lesion					3.20	0.20
T1a	36	14	22	38.9		
T1b	36	13	23	36.1		
T2-3	28	16	12	57.1		
Lymph node metastasis					15.10	0.00**
Yes	43	28	15	65.1		
No	57	15	42	26.3		

* P<0.05;

** P<0.01, indicating that TS expression is associated with clincal stage and a history of smoking.

TS, thymidylate synthase.
